# Delayed Operative Management of Fractures of the Lateral Condyle of the Humerus in Children

**DOI:** 10.5704/MOJ.1503.010

**Published:** 2015-03

**Authors:** AD Shabir, AD Tahir, AW Sharief, HD Imtiyaz, H Shahid, AD Reyaz

**Affiliations:** SKIMS Medical College Bemina, Srinagar, Kashmir India

**Keywords:** Lateral condyle, delayed, surgical management, paediatric

## Abstract

**Purpose:**

Delayed presentation of lateral condylar fractures of the humerus is relatively common in the developing regions of the world. These fractures are difficult to manage because of the displacement and fibrosis around the condylar fragment secondary to the delay. There is a paucity of literature concerning the management of these fractures. An oft repeated finding is the requirement of extensive dissection around the fragment for proper reduction. The purpose of this study was to assess the efficacy of surgical management of lateral condylar fractures with delayed presentation.

**Methods:**

We assessed the results of lateral condylar fracture fixation in 20 cases with delayed presentation.

**Results:**

The lateral condylar fractures in patients with a delayed presentation can be managed surgically with good results.

**Conclusions:**

Open reduction and internal fixation should continue to be the method of choice for the management of lateral condylar fractures which report late for management.

## Introduction

Fractures of the lateral condyle of the humerus comprise 18.5 per cent of all fractures of the distal end of the humerus in children^1^. The incidence of the functional loss of the range of motion of the elbow is much greater with fractures of the lateral condylar physis because the fracture line extends into the articular surface. A poorly treated fracture of the lateral condyle is likely to result in significant loss of range of motion of the elbow that is not as responsive to surgical correction. The complications of lateral condylar physis may not be obvious months after the initial injury.

There is a consensus in literature about the requirement of early intervention and anatomical reduction of these fractures with Dhillon *et al* and Zionts *et al* reporting uniformly bad results which included cubitus varus and valgus deformities, osteonecrosis, nonunion and malunion, and loss of motion. They recommended that patients presenting late be left alone and any sequelae evaluated at a late stage^2,3,4^. Fractures that are operated upon after a delay are also complicated by the presence of fibrosis, and callus formation. Preoperative stiffness that is found in these cases is likely to affect the post operative result^3^.

We report the results of operative management of twenty lateral condyle fractures of the humerus in the paediatric population that presented after more than 3 weeks of the initial trauma.

## Materials and Methods

20 patients ranging from 3 years to 13 years were included in the study. All patients presenting after more than 3 weeks of trauma were included in this prospective study. There were 12 boys and 8 girls. The fractures were difficult to classify radiologically in view of the delay. However an assessment was still attempted.

### Surgical technique

A lateral approach was made to the elbow. Dissection through the plane between the triceps and the brachioradialis. The approach was carried through the lateral fascia right down to the fracture. The fragment was often found to be displaced and fibrous tissue often made it difficult to assess the orientation of the fracture. Careful dissection of the fibrous tissue was made and posterior attachments were saved. Thorough irrigation was done to remove the fibrinous debris. Any dissection needing to be done on the lateral epicondyle and metaphysis was made anterior, to avoid the posterior blood supply and minimize the risk for avascular necrosis. The displaced fragment was reduced under direct visualization, often with the aid of a reduction clamp, “joystick” Kirschner (K)‐wires, or the assistant's manual pressure. We used 1.2-mm-diameter Kirschner wires for patients younger than five years of age, 1.4-mm-diameter wires for those between five and eight years of age and 1.8-mm diameter wires for those older than eight years of age. Grafting was not added in any patient. Post operatively all patients underwent a common protocol of 3 weeks padded cramer wire splint immobilisation followed by range of motion exercises intermittently for a further 2 weeks. At 4 weeks the k wire was removed. The patient was allowed to do range of motion exercises without splint protection at 5 weeks.

All patients were followed up for a period of one year. At one year the final assessment was done on the basis of the Hardacre criteria^5^. Radiologically avascular necrosis, malunion, non-union and heterotropic ossification were specifically looked at.

Union was assessed on the anteroposterior and lateral radiographs of the elbow. Union was said to have occurred when the fracture was obliterated by the trabeculae or the callus.

## Results

20 patients ranging from 3 years to 13 years were included in the study. All patients presenting after more than 3 weeks of trauma were included in the study. There were 12 boys and 8 girls. The fractures were difficult to classify radiologically in view of the delay. However an assessment was still attempted. There were 4 fractures which matched the Milch type 1. All the others were classified as Milch type 2.

The average delay in the presentation was 6.2 weeks [3-16 weeks]. 8 cases had visited local bone setters initially, 6 had not consulted anyone and tried home remedies, 4 patients had displaced in the plaster splint and 2 had been missed by the treating doctors.

At final follow up,the mean carrying angle in the fractured elbow was 6 degrees (range from 0 to 12 degrees), and 7degrees (range from 5 to 10 degrees) in the other elbow.

The range of motion improved by an average of 60 degrees from an average of 45 degrees preoperatively to 105 degrees postoperatively.

Union occurred in 18 cases from 6‐24 weeks with an average of 10 weeks.

According to Hardacre criteria (Table 1), the functional results were excellent in 7 fractures, good in 10 fractures and poor in 3. The poor results had a persistent non union in 2 cases and significant stiffness in one. The patient with significant stiffness was not compliant with the physiotherapy protocol. Both non union patients had needed more dissection at surgery due to excessive fibrosis. Lateral humeral condyle was clinically and radiographically more prominent in 12 patients due to formation of new and extra bone. Review of early radiographs demonstrated that the bone spicules were elevated from the lateral condyle. The spicules were probably osteoperiosteal flaps, and the lateral prominence was depending on the formation of bone between spicule and lateral condyle.

**Table 1:** Evaluation of treatment outcomes in humeral lateral condyle injuries with Hardacre criteria^5^

Excellent	Full range of motion Normal carrying angle and appearance No symptoms Complete healing of fracture
Good	Efficient range of motion Loss of extension less than 15 degrees Mild and subtle deformity No arthritic or neurological symptoms Complete healing of fracture
Fair	Loss of motion to the extent of disability Alterations in carrying angle and prominent deformity Presence of arthritic or neurological symptoms Presence of nonunion or avascular necrosis

It can be appreciated that due to the unavailability of such patients in large numbers, the statistical analysis is constrained by the small size of the series.

## Discussion

Fractures of the lateral condyle of the humerus are amongst the commonest injuries encountered by the orthopaedic surgeon. Henry Milch differentiated between the two fractures patterns. A fracture exiting the trochleocapitellar groove as type 1 and the fracture exiting the trochlea as type 2^6^. Most trauma the degree of displacement depends on the preservation of the articular hinge. If the hinge is intact the condylar fragment shows only a lateral tilt. If the fracture is complete the fracture can be rotated completely up to almost 180 degrees.

Whilst there is a consensus about the management of fractures treated early, fixation of delayed fractures is surrounded by confusion. A late presentation leads to difficulty in management due to displacement of the fragment as a result of the pull of the common extensors, incongruous reduction of articular surfaces, injury/early closure of the epiphyseal growth plate, and possible damage to vascular supply.

Lagrange and Rigault showed that the blood supply to the lateral condyle enters by its soft‐tissue attachments, particularly posteriorly at the origin of the long extensor muscles, and disruption of this will destroy the vessels and render the condyle ischaemic^7^.

**Fig. 1:**
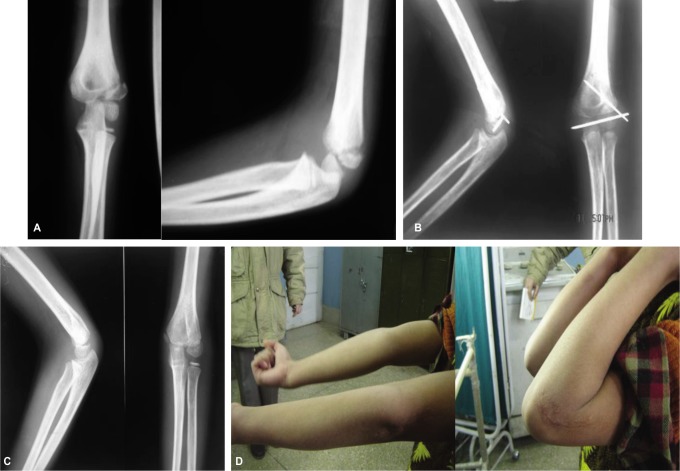
Showing the lateral condyle fracture in an 8 year old boy [A]. Open reduction and fixation was done after 6 weeks [B]. Final radiograph and clinical range of motion at 1 year [C and D].

**Fig. 2:**
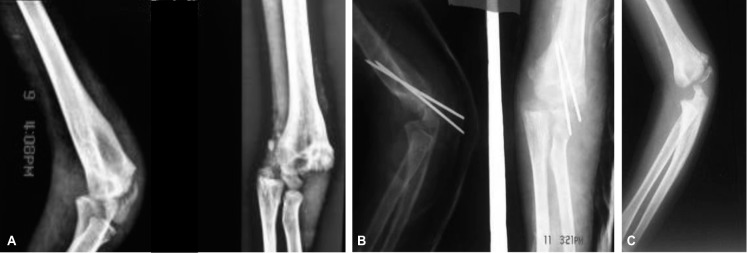
The AP view of Fig. 2 appears to be a fracture‐separation of the distal humeral physis and not a lateral condyle fracture.

Surgical results in patients operated after delays are reported to be uniformly bad. In a series of seven cases operated more than 2 weeks after the injury, Jakobs *et al* reported uniformly bad results. They reported non union, malunion, persistent subluxation, limitation of motion and avascular necrosis^8^.

The surgical technique should not be too aggressive to disturb the condylar vascularization. In order to control the intra-articular reduction, it may be necessary to cut some parts of the capsule and the synovia^9,10^. Jakob *et al* felt that surgical intervention in cases with delayed presentation did not improve results in comparison to patients with no treatment at all^8^. They pointed out the difficulty caused by the early callus formation. Whilst studies aim at a less than 2mm step at the articular cartilage, the criteria for cases with delayed presentation are not defined^11^.

**Fig. 3:**
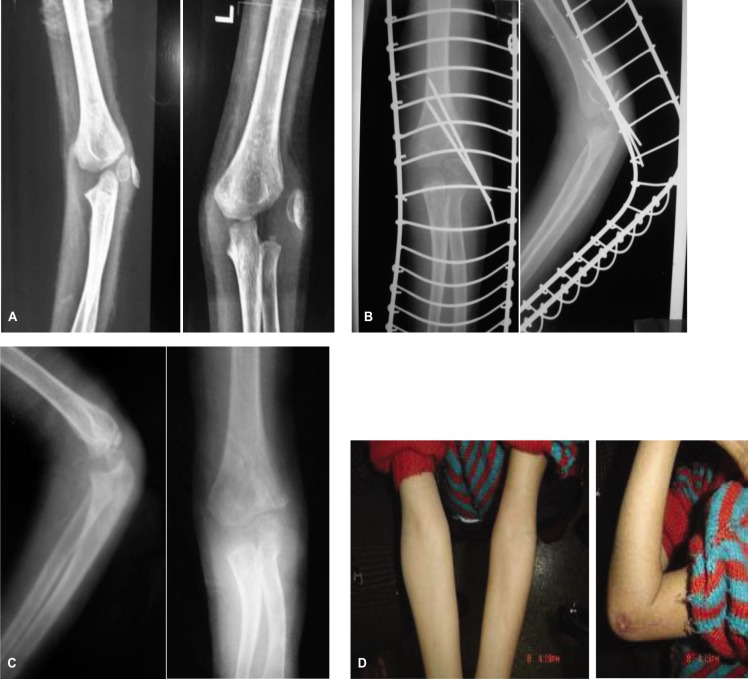
8 week old fracture of the lateral condyle [A]. Immediate post operative and final x rays [B and C]. Final range of motion is also depicted [D].

There is a general agreement that surgical intervention in old established non unions should be avoided as osteosynthesis may reduce the range of motion of the elbow or the bone may not unite, so operative treatment for such patients has not been popular^12,13^.

However studies differing from this concept have reported reasonable results after proper patient selection^14^. According to Toh *et al* nonunions consistently lead to pain, instability, loss of function, and tardy ulnar nerve palsy, they should be treated as soon as possible after injury, preferably before skeletal maturity^15^.

Aggarwal *et al* reported in their study of 22 cases with delayed presentation. According to them exact anatomical reduction of the lateral condylar fragment were difficult to achieve, but conspicuous alteration in carrying angle was not present except in 2 cases. Fish-tail appearance was seen in 7 cases and premature closure of lateral condylar epiphysis was noted in 4 cases^16^.

Saraf *et al* in their series of 20 cases reported avascular necrosis of the lateral condyle in one patient, premature fusion in two patients, pin tract infection in three patients, and gross restriction of elbow movements in three patients^17^.

Both series report union times similar to ours.

All cases in our series reported for treatment after more than 3 weeks of the injury. Expectedly all our cases had significant restriction of motion at presentation. It was important that posterior dissection be avoided as the possibility of avascular necrosis would be high.

Our series supports the view that lateral condylar fractures with a delayed presentation in spite of being more difficult to fix than fresh fractures can still be managed operatively with good results.
